# Galcanezumab for the treatment of chronic migraine and medication overuse headache: Real‐world clinical evidence in a severely impaired patient population

**DOI:** 10.1002/brb3.2799

**Published:** 2023-05-19

**Authors:** Simona Guerzoni, Carlo Baraldi, Flavia Lo Castro, Maria Michela Cainazzo, Luca Pani

**Affiliations:** ^1^ Digital and Predictive Medicine, Pharmacology and Clinical Metabolic Toxicology‐Headache Center and Drug Abuse‐Laboratory of Clinical Pharmacology and Pharmacogenomics, Department of Specialist Medicines AOU Policlinico di Modena Modena Italy; ^2^ PhD School in Neurosciences, Department of Biomedical, Metabolic and Neural Sciences University of Modena and Reggio Emilia Modena Italy; ^3^ Post‐Gradute School in Pharmacology, Department of Biomedical, Metabolic and Neural Sciences University of Modena and Reggio Emilia Modena Italy; ^4^ Pharmacology Unit, Department of Biomedical, Metabolic and Neural Sciences University of Modena and Reggio Emilia Modena Italy; ^5^ Department of Psychiatry and Behavioral Sciences University of Miami Miami Florida USA; ^6^ VeraSci Durham North Carolina USA

**Keywords:** chronic migraine, galcanezumab, headache days, medication overuse‐headache, preventive treatment

## Abstract

**Background:**

Galcanezumab is a monoclonal antibody acting against the calcitonin gene‐related peptide approved for the preventive treatment of migraine. The aim of this article is to explore its effectiveness and safety of galcanezumab in chronic migraine (CM) with medication overuse‐headache (MOH).

**Methods:**

Seventy‐eight patients were consecutively enrolled at the Modena headache center and followed up for 15 months. Visits were scheduled every 3 months, and the following variables were collected: the number of migraine days per month (MDM); the painkillers taken per month (PM); the number of days per month in which the patient took, at least, one painkiller; the six‐item headache impact test; and the migraine disability assessment questionnaire (MIDAS) score. Demographic features of the analyzed sample were collected at the baseline and adverse events (AEs) were collected at every visit.

**Results:**

After 12 months, galcanezumab significantly reduced the MDM, the PM, the number of days on medication, the HIT‐6 as well as the MIDAS scores (all *p* < .0001). The greatest amelioration was obtained in the first trimester of treatment. A higher MDM, a higher NRS score at the baseline, and a higher number of failed preventive treatments negatively predict the CM relief at the year of treatment. No serious AEs were registered and only one drop‐out was due to AE.

**Conclusions:**

Galcanezumab is effective and safe for the treatment of patients affected by CM and MOH. Patients with a higher impairment at the baseline may found less benefits with galcanezumab.

## Graphical abstract

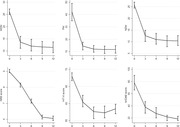

## 1 INTRODUCTION

Migraine is characterized by attacks of unilateral, pulsating, excruciating head pain, often associated with nausea, vomiting, photophobia, and/or phonophobia (Headache Classification Committee of the International Headache Society [IHS], 2018). Chronic migraine (CM) is defined as the recurrence of migraine attacks for ≥15 days per month for, at least, 3 months (Headache Classification Committee of the International Headache Society [IHS] 2018). CM sufferers usually take a large number of painkillers to treat the recurrent attacks, and this may generate a secondary headache called medication overuse‐headache, thus worsening CM (Headache Classification Committee of the International Headache Society [IHS], 2018; Sun‐Edelstein et al., 2021). CM complicated with medication overuse‐headache (MOH) has a prevalence of about 1%–2% of the general population in western countries (Burch et al., 2019) and represents a significant burden for society (Lanteri‐Minet et al., 2011). Indeed, the recurrent pain strongly limits patients in performing their social and working activities, thus potentially leading to working impairment and social exclusion, even followed by the development of anxiety and depression (Nielsen et al., 2019). Additionally, until the approval of the new drugs acting upon the calcitonin gene‐related peptide (CGRP) pathway, CM complicated with MOH was the most refractory form of headache, and a painkiller withdrawal was necessary before a new preventive treatment could be started (Nielsen et al., 2019). Anti‐CGRP monoclonal antibodies (mAbs) are well tolerated and effective drugs for CM, even without a drug withdrawal (Pensato et al., 2022). In particular, galcanezumab is an anti‐CGRP mAb that has shown promising efficacy and safety in treating CM in randomized placebo‐controlled trials (Detke et al., 2018). Additionally, galcanezumab showed a good efficacy even in patients with previous preventive treatment failures (Ruff et al., 2019), showing a stable efficacy over time (Födrerreuther et al., 2018; Pozo‐Rosich et al., 2022). Notably, galcanezumab showed a good efficacy even in patients affected by CM and MOH (Dodick et al., 2021). Despite this, no studies have explicitly focused on galcanezumab action upon in real‐word settings, CM and MOH, even if some evidence suggests a positive action of galcanezumab even in this condition (Vernieri, Altamura, et al., 2021). Hence, this article aims to explore the effectiveness and safety of galcanezumab in a cohort of patients affected by CM and MOH after 1 year of therapy. Moreover, the clinical course of patients after the suspension of galcanezumab has also been explored. Finally, potential predictors of the response to galcanezumab have been searched.

## 2 MATERIALS AND METHODS

### 2.1 Study design and ethical approval

This single‐center, prospective study was conducted at the University hospital of Modena. All participants signed an informed consent to participate in the research and for data publication. This study was approved by the Area Vasta Emilia Nord Ethics Committee of Modena (protocol number: 625/2020/OSS/AOUMO) and was conducted in accordance with the latest version of the Declaration of Helsinki.

### 2.2 Participants and data collected

Patients affected by CM and MOH according to the International Classification of Headache Disorders‐3rd Edition (ICHD‐3) (Headache Classification Committee of the International Headache Society [IHS], 2018) and followed up in the headache center of the University of Modena were consecutively enrolled between the November 1, 2020 to the May 1, 2021 during one of their scheduled visits at the center, when galcanezumab was prescribed. Afterward, patients received galcanezumab subcutaneously every 30 days for up to 12 months, according to the EHF guidelines (Födrerreuther et al., 2018) and the European Medicines Agency (EMA) summary of product characteristics (https://www.ema.europa.eu/en/documents/product‐information/aimovig‐epar‐product‐information_en.pdf). All patients received a dose of galcanezumab of a vial of 120 mg subcutaneously every month, except for the first injection when a double loading dose was administered. After reaching the year of treatment, galcanezumab was discontinued (Sacco et al., 2019) and the patients were reevaluated after 3 months. The drug was restarted in case of migraine worsening (Sacco et al., 2019). Visits were scheduled every 3 months. The following variables were collected: the average number of migraine days per month (MDM), the average number of painkillers taken per month (painkillers per month, PM), the mean number of days per month in which the patient took, at least, one painkiller (number of days on medication, NDM), the average migraine intensity using the numeric rating scale for pain (NRS) score, the six‐item headache impact test (HIT‐6) score (Yang et al., 2011) and the migraine disability assessment questionnaire (MIDAS) score (Bigal et al., 2003), and the hospital anxiety and depression scale (HADSd for depression and HADSa for anxiety) score (Bjelland et al., 2022). The following variables were collected only at the baseline visit: age, sex, familiarity with headache, presence of migraine aura, duration of migraine, duration of CM, the number of preventive treatments failed in the past, the concomitant assumption of another preventive medicine, and the performing of an in‐hospital drug withdrawal before starting galcanezumab. Adverse events (AEs) were collected at every visit and analyzed. Local adverse events related to the injection procedure were considered only if lasted for more than 24 h.

### 2.3 Statistical analysis

Continuous variables were checked for normal distribution using the Shapiro–Wilk test and compared with the one‐way analysis of variance followed by Sidak's test for multiple comparisons if normally distributed. Otherwise, a Kruskal–Wallis rank‐sum test was used. Categorical variables were expressed as subject counts and percentages and compared using the chi‐squared test for homogeneity of odds. The variables collected at the baseline were compared between those patients who were still suffering from CM after 12 months of treatment and those who were not suffering from CM after 12 months of treatment. In particular, a logistic univariate analysis was performed first, followed by multiple logistic regression analyses with backward elimination for those variables significantly associated with CM and MOH at the univariate level. The model was tested for collinearity using the phi correlation coefficient, and collinear variables were eliminated from the model. The Pearson's *χ* ^2^ goodness of fit test was carried out to assess the goodness of fit of the entire model. The receiver operating characteristic curve (ROC) analysis was performed upon the whole model. Sample size calculation was based upon the results obtained by an article written by Pozo‐Rosich's group (Pozo‐Rosich et al., 2022), considering the total decrease of the number of migraine days after 12 months of treatment, with a power of 80% and an alpha error of 5%. *p*‐Values lower than .05 were considered significant. Statistical calculations were made with the STATAIc 15.1 software.

## 3 RESULTS

### 3.1 Demographic features

Globally, 78 patients were enrolled. The analyzed sample was mainly composed of middle‐aged women with a long duration of CM and MOH. Moreover, the present sample displayed a high number of previous preventive treatments failures and an almost‐daily migraine and analgesic consumption. Demographic features are summarized in Table 1.

**TABLE 1:** Demographic features of the analyzed sample

Variable	Value
Patients	78 (100%)
Age	51.63 ± 8.9
Female sex	58/75 (77.33%)
Aura	5/75 (6.67%)
Familial aggregation of migraine	53/75 (76.81%)
Duration of migraine	34.69 ± 12.16
Duration of CM	13.80 ± 10.92
Duration of MOH	11.8 ± 9.82
Anxiety (HADSa > 11)	8/75 (10.67%)
Depression (HADSd > 11)	15/75 (20%)
Comorbidities	36/75 (48%)
Psychiatric comorbidities	20/75 (26.67%)
Gastrointestinal comorbidities	20/75 (26.67%)
Cardiovascular comorbidities	14/75 (18.67%)
Endocrinological comorbidities	8/75 (10.67%)
Ginecological comorbidities	5/75 (6.67%)
Respiratory comorbidities	4/75 (5.33%)
Neurological comorbidities	1/75 (1.33%)
Preventive treatment failed	5.64 ± 2.49
Previous drug withdrawal	7/75 (9.33%)
Preventive treatment other than galcanezumab	26/75 (34.67%)
MDM	26.03 ± 5.04
PM	41.96 ± 30.56
NDM	25.72 ± 5.58
NRS score	8.88 ± 0.66
HIT‐6 score	67.83 ± 4.9
MIDAS score	77.97 ± 51.33

Abbreviations: CM, chronic migraine; MOH, medication overuse‐headache; HADS, hospital anxiety and depression scale; HIT‐6, six‐item headache impact test; MDM, migraine days per month; MIDAS, migraine disability assessment questionnaire; NDM, number of days on medication; NRS, numeric rating scale; PM, painkillers per month.

### 3.2 Changes in the MMD, PTPM, NDM, NRS score, HIT‐6 score, MIDAS score, and the HADAS scores under galcanezumab treatment

**FIGURE 1:**
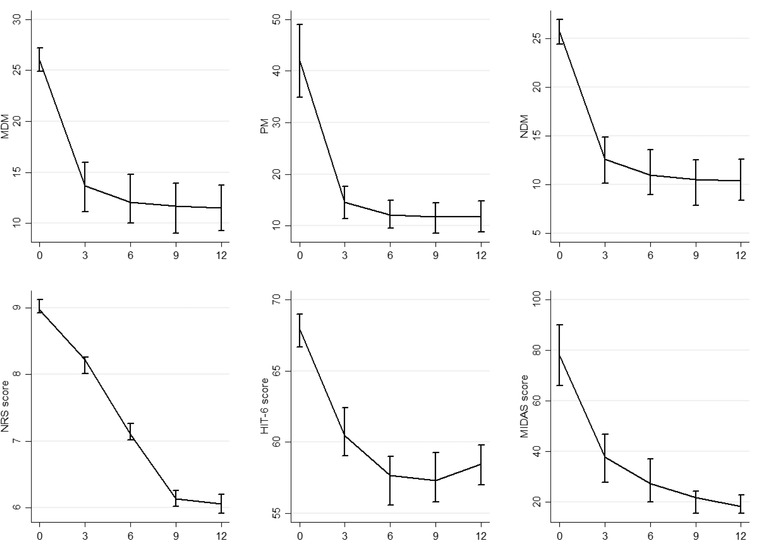
Migraine days per month (MDM), painkillers per month (PM), number of days on medication (NDM), numeric rating scale for pain (NRS) score, six‐item headache impact test (HIT6) score, and migraine disability assessment questionnaire (MIDAS) score at every month

**FIGURE 2:**
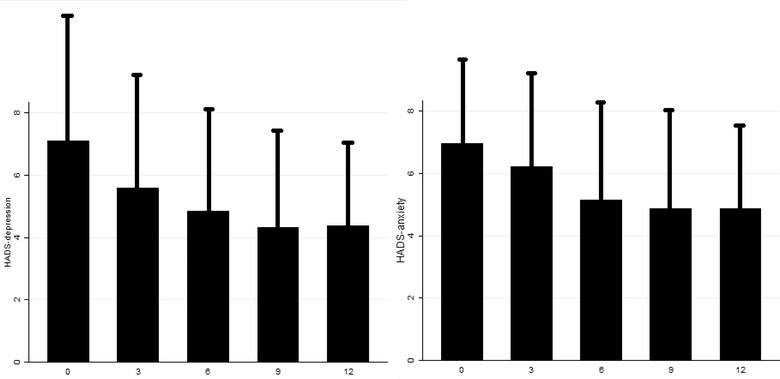
HADSa and HADSd at every month

When compared to the baseline, the MDM was significantly reduced after 3 months (13.55 ± 10.35), 6 months (12.41 ± 10.03), 9 months (11.46 ± 9.34), and 12 months (11.5 ± 8.99) (all *p* < .001). Moreover, the PM significantly reduced to 14.5 ± 13.26 in the 3rd month, 12.23 ± 11.43 in the 6th month, 11.53 ± 11.37 in the 9^th^ month, and 11.81 ± 12.06 after 1 year (*p* < .001). The NDM reduced to 12.53 ± 10.08 after 3 months, to 11.28 ± 9.81 after 6 months, to 10.2 ± 8.95 after 9 months, and to 10.5 ± 8.52 after 12 months (all *p* < .001). The NRS score significantly reduced compared to the baseline, with a total decrease of −2.83 ± 0.04 after 12 months. The HIT‐6 and the MIDAS scores reduced to 58.4 ± 5.58 and 19.26 ± 14.51 after 12 months, respectively (all *p* < .001). These data are graphically summarized in Figure 1. The HADSd and the HADSa significantly reduced trough time: from the 6th month onward, the HADSd resulted significantly lower than the baseline, so did the HADSa. In particular, the HADSd decreased to 5.57 ± 3.67 at the 3rd month (*p* = .08), to 4.85 ± 3.43 (*p* = .001) at the 6th month, to 4.33 ± 3.08 (*p* < .001) at the 9th month, and to 4.37 ± 2.47 (*p* < .001) at the year of treatment. The HADSa reduced to 6.23 ± 3.24 after 3 months (*p* = .817), to 5.13 ± 3.12 after 6 months (*p* = .005), to 4.87 ± 3.26 after 9 months (*p* = .001), and to 4.86 ± 2.66 (*p* = .001) after 1 year of treatment. These results are summarized in Figure 2.

### 3.3 Predictors of CM relief after 12 months of treatment

In the univariate analysis, patients who were still CM sufferers after 1 year of treatment displayed the following: a higher number of MDM at the baseline (28.64 ± 2.78 vs. 24.47 ± 5.45, *p* = .002), a higher number of PTPM at the baseline (56.93 ± 31.04 vs. 33.04 ± 26.82, *p* = .004), a higher NDM at the baseline (28.46 ± 3.16 vs. 24.09 ± 6.08, *p* = .003), a higher NRS score at the baseline (9.14 ± 0.45 vs. 8.72 ± 0.71, *p* = .014), a higher number of preventive treatment failed in the past (6.57 ± 3.02 vs. 5.09 ± 1.93, *p* = .021), and a higher MIDAS score at the baseline (97.39 ± 70.54 vs. 65.89 ± 29.47, *p* = .022). For the multivariate analysis, independent predictors of CM relief after 1 year were as follows: a lower MMD at the baseline (*p* = .023), a lower NRS score at the baseline (*p* = .038), and a lower number of failed preventive treatments in the past (*p* = .043). These data are summarized in Table 2. The model fitted well (Pearson's *χ* ^2^ = 58.5, *p* = .761), and the ROC curve displayed an area under the curve of 0.8413.

**TABLE 2:** Comparison between chronic migraine (CM) sufferers after 1 year and the ones who became episodic migraineurs

	Status at the 9th month		Univariate analysis		Multivariate analysis	
Baseline characteristics	Episodic migraineurs 47/75 (62.67 %)	Chronic migraineurs 28/75 (37.33%)	*p*‐Value	OR (95% CI)	*p*‐Value	OR (95% CI)
Age	50.40 ± 8.33	53.68 ± 9.71	.13	–	–	–
Female sex	34/47 (72.34 %)	24/28 (85.71%)	.188	2.29 [0.67–7.9]	–	–
Migraine duration	34.45 ± 11.52	35.11 ± 13.52	.82	–	–	–
CM duration	12.87 ± 10.25	15.32 ± 12.12	.352	–	–	–
MOH duration	93.54 ± 69.06	115.64 ± 90.1	.247	1 [0.99–1.01]		
Aura	3/47 (6.38 %)	2/28 (7.14 %)	.851	1.19 [0.19–7.65]	–	–
Anxiety	7/47 (14.89 %)	1/28 (3.87 %)	.157	0.21 [0.02–1.82]	–	–
Depression	12/47 (25.53%)	3/28 (10.71%)	.132	0.35 [0.09–1.37]	–	–
MDM	24.47 ± 5.45	28.64 ± 2.78	.002	–	.023	1.22 [1.03–1.44]
PM	33.04 ± 26.82	56.93 ± 31.04	.004	–	.576	1.01 [0.98–1.03]
NDM	24.09 ± 6.08	28.46 ± 3.16	.003	–	Dropped due to collinearity with MD	
NRS score	8.72 ± 0.71	9.14 ± 0.45	.014	–	.038	3.33 [1.07–10.41]
Number of failed prophylaxis	5.09 ± 1.93	6.57 ± 3.02	.021	–	.043	1.36 [1.01–1.83]
Detoxification	3/47 (6.38 %)	4/28 (14.29%)	.267	2.44 [0.5–11.84]	–	–
Galcanezumab in add‐on	13/47 (27.66 %)	13/28 (46.43 %)	.102	2.27 [0.85–6.04]	–	–
MIDAS	65.89 ± 29.47	97.39 ± 70.54	.022	–	.366	1.01 [0.99–1.02]
HIT‐6	67.09 ± 4.84	69 ± 4.85	.112	–	–	–

Abbreviations: CI, confidence interval; MOH, medication overuse‐headache; HADS, hospital anxiety and depression scale; HIT‐6, six‐item headache impact test; MDM, migraine days per month; MIDAS, migraine disability assessment questionnaire; NDM, number of days on medication; NRS, numeric rating scale; OD, odds ratio; PM, painkillers per month.

### 3.4 Adverse events and drop‐outs

Twenty‐eight patients suffered from, at least one AE, and the percentage of AEs for each cycle was as follows: 25.33% (19 patients) in the first 3 months, 21.33% (16 patients) from the 3rd to the 6th months of treatment, 20% from the 6th to the 9th months (15 patients), and 22.67% (17 patients) in the last 3 months before the end of the year of treatment. The proportion of patients reporting an adverse event did not differ between the visits (*p* = .8802). All registered AEs are summarized in Table 3. Globally, three dropouts were registered. In particular, two patients were lost at follow‐up before the 3rd and 6th month, while one patient had to suspend treatment because he suffered from severe low back pain before reaching the 3rd month of treatment.

**TABLE 3:** Adverse events

Adverse event	0–3 Months	3–6 Months	6–9 Months	9–12 Months
Constipation	13	13	13	16
Fatigue	1	1	0	0
Vertigo	2	1	0	0
Injection site redness	2	1	1	1
Injection site itching	0	0	1	0
Low back pain	1	0	0	0

### 3.5 Changes in the MDM, PM, NDM, NRS score, HIT‐6 score, and MIDAS score during the suspension of galcanezumab

**FIGURE 3:**
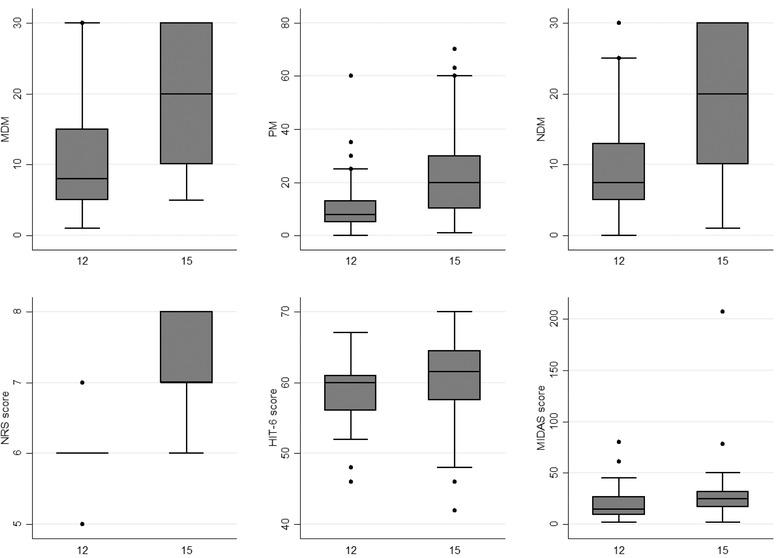
Migraine days per month (MDM), painkillers per month (PM), number of days on medication (NDM), numeric rating scale for pain (NRS) score, six‐item headache impact test (HIT6) score, and migraine disability assessment questionnaire (MIDAS) score before and after the discontinuation of galcanezumab

Twenty patients were reevaluated after the 3‐month scheduled suspension of galcanezumab beyond the year of treatment. These patients displayed an increase in the MDM (18.55 ± 9.1, *p* = .0008), PM (25.35 ± 20.64, *p* = .0007), NDM (17.85 ± 9.89, *p* = .0013), NRS score (6.65 ± 0.81, *p* = .0526), HIT‐6 score (60.05 ± 7.29, *p* = .0874), and MIDAS score (35.35 ± 43.46, *p* = .0223). These data are summarized in Figure 2. The HADSa did not change (6.8 ± 4.8, *p* = .0855) and the HADSd (7.05 ± 4.72, *p* = .0167) slightly increased. However, all the explored parameters resulted significantly improved if compared to the baseline. Among the other 55 patients, 10 were lost at follow‐up, and the others were waiting for the visit after the treatment stop (Figure 3).

## 4 DISCUSSION

The present study confirmed the effectiveness of galcanezumab even in a severely impaired population of CM and MOH sufferers, which are notoriously the most refractory to the preventive treatments for migraine (Vandenbussche et al., 2020). Furthermore, the analyzed sample displayed an almost‐daily migraine frequency at the baseline; patients had to take an average number of even more than one painkiller per day to treat their attacks and had failed a high number of preventive treatments in the past. The significant impairment at the baseline was summarized by the MIDAS score, which indicated a severe impact of migraine on patients’ quality of life. Despite this, after the first 3 months of treatment, patients already displayed a significant reduction of the MDM, PM, NDM, HIT‐6, and MIDAS scores compared to the baseline (Figure 1). This result also confirms in a population of CM and MOH sufferers the rapid onset of action of galcanezumab, which has been already assessed among episodic migraineurs (Iagarshi et al., 2021) as well as CM sufferers who had failed multiple preventive medications (Schwedt et al., 2021), with a clinically meaningful improvement even after the first week of treatment (Kuruppu, North, et al., 2021). Moreover, the improvement in patients’ disability has been specifically assessed in CM sufferers (Ford et al., 2021). However, despite the habitual rapid onset of action of galcanezumab, not all patients experience a rapid response (Goadsby et al., 2019). However, the 50% of patients who do not respond after the first and second months may respond within the 3rd month, at least in episodic migraine (Goadsby et al., 2019). Hence, a follow‐up of at least 3 months is warranted. The rapid onset of action of galcanezumab is linked to the early reach of the peak serum (5 days) (Kuruppu, North, et al., 2021) and the initial administration of a double loading dose of 240 mg instead of the 120 mg dose used for the other injections. About the last point, it has been proved that the reduction of the free CGRP is dose‐ and time‐dependent for galcanezumab, with a higher dose determining a higher reduction (Kielbasa & Helton, 2019). This could also explain why, after the great improvement in the first 3 months of treatment, the effect of galcanezumab stabilizes over time (Pozo‐Rosich et al., 2022), thus ruling out the existence of a tolerance effect. In this sample, an amelioration of anxious and depressive symptoms was observed. Smitherman et al. (2020) indicated that CM sufferers with comorbid anxiety and depression responded to the 240 mg dose but not to the 120 mg one, thus necessitating additional interventions. In our study, patients were screened for anxious and depressive symptoms using the HADS score, but a few scored more than 11 points, the threshold for mild depression and anxiety (Bjelland et al., 2022). Due to this, our results are poor compared to the ones obtained by Smitherman et al. (2020) but should be taken as a proof of the effectiveness of galcanezumab over anxious and/or depressive symptoms. Interestingly, while the effects of galcanezumab on migraine features spread within the first 3 months, the HADSa and HADSd scores ameliorated after that time point, that is, after the 4th injection. This may indicate that this effect is mainly linked to the migraine relief rather than a direct action of anti‐CGRP mAbs on anxiety and depression. Indeed, galcanezumab has a peripheral site of action, and the peripheral CGRP pathway has a marginal importance in the pathogenesis of the psychiatric comorbidities of CM and MOH (Goadsby et al., 2019; Hashikawa‐Hobara et al., 2021). Despite the good effectiveness observed in this study, 28 patients still suffered from CM at the end of the year of treatment. So, the predictors of CM relief after 1 year of treatment were explored. A greater impairment at the baseline was associated with a lower response toward galcanezumab (Table 2). In particular, a higher MDM, a higher NRS score at the baseline, and a higher number of failed preventive treatments in the past were independent predictors of CM persistence after 1 year of treatment. A higher number of migraine days per month has been associated with a lower response to anti‐CGRP mAbs in CM (Caronna et al., 2021; Iannone et al., 2022). Moreover, in a similar population from our center, a higher number of failed preventive treatments were associated with a lower response toward erenumab (Baraldi et al., 2021). Pain intensity has not been linked to the response to the anti‐CGRP mAbs, but pain catastrophizing has been linked to the response to the anti‐CGRP mAbs (Silvestro et al., 2021). Many biochemical mechanisms may explain a lower response to the anti‐CGRP mAbs and occur peripherally since anti‐CGRP mAbs do not pass the blood–brain barrier (BBB) (Kielbasa & Helton, 2019). In particular, galcanezumab binds the 61% of the free CGRP at the steady state (Kielbasa & Helton, 2019). Hence, a considerable amount of the free CGRP may be able to bind and activate its receptors. Additionally, due to the calcitonin peptide family, other peptides, such as amylin, adrenomedullin‐1, and adrenomedullin‐2, may bind to the CGRP receptors and activate them, even if less potently (Edvinson et al., 2022). On the other hand, the abovementioned peptides may activate their receptors and offset the blocking effect of anti‐CGRP mAbs on CGRP signaling (Rees et al., 2022). Considering this, how can it be biologically explicable that the MDM, the pain intensity, and the preventive treatment failed in the past influence the response to anti‐CGRP mAbs? It has been proven that CM complicated with MOH is associated with higher CGRP levels in the peripheral blood, which restore after the successful withdrawal of the overuse of painkillers (Greco et al., 2020). Hence, it is not unreasonable to think that a higher MDM at the baseline may be associated with higher CGRP levels and, consequently, a lower response to anti‐CGRP mAbs. These data may also explain why in our previous article, the number of migraine days per month was not associated with the response to erenumab, since it acts on the CGRP receptor rather than on the ligand (Sacco et al., 2019). Pain intensity is associated with higher CGRP levels in somatic pain (Schou et al., 2017), but no study has linked higher CGRP levels with the intensity of a migraine attack. It could be possible that the recurrence of high‐intensity attacks may determine higher CGRP levels. Furthermore, the failure of many preventive treatments in the past may indicate a more resistant form of migraine. Even if this category displays a worse response to anti‐CGRP mAbs, they respond well (Kuruppu, Tobin, et al., 2021). A study from Tassorelli's group demonstrated that patients with anxiety disorders are less likely to respond to anti‐CGRP mAbs (Bottiroli et al., 2021), confirming the results obtained in another study among CM sufferers treated with galcanezumab in 2020 (Smitherman et al., 2020). Pain and depression are processed by the same brain areas, thus determining a possible overlapping between the two conditions (Zheng et al., 2022), and this may explain why the two conditions are often correlated. Moreover, stating the incapability of the anti‐CGRP mAb to cross the BBB and to reach the abovementioned areas, it should be arguable that depressive symptoms may be refractory toward anti‐CGRP mAbs (Irimia et al., 2021). In our study, we did not find an independent effect of anxiety or depression on the response to galcanezumab. Still, the percentage of patients suffering from anxiety and depression was meager at the baseline not allowing a reliable comparison. After 1 year of treatment, the three‐month suspension determined a worsening in the MDM, PM, NDM, NRS score, HIT‐6, and MIDAS score. This confirmed that the data indicate the worsening of migraine after 3 months of suspension (Raffaelli et al., 2022; Vernieri, Brunelli, et al., 2021). It seems that migraine worsening after the suspension of galcanezumab spread slower than with erenumab, probably because of the higher half‐life of the first mAb (27 vs. 21 days) (Kielbasa & Helton, 2019). Migraine worsening indicates that, despite their good effectiveness, anti‐CGRP mAbs are not disease‐modifying agents (Vernieri, Brunelli, et al., 2021). Some oral preventive treatments have been shown to reduce the number of migraine days per month for several months after treatment cessation (Diener et al., 2007; Wöber et al., 1991). The explanation of this prolonged effect may be searched in the central action of these drugs. On the contrary, galcanezumab acts outside the BBB (Kielbasa & Helton, 2019), so it can only lead to peripheral neuronal network changes that poorly interfere with the central mechanisms of pain chronicity, that is, central sensitization (Mungoven et al., 2021). Interestingly, despite the worsening, patients resulted less impaired after the 3‐month stop compared with the baseline. This indicates that a 3‐month period is not enough to lose all the benefit of anti‐CGRP mAbs, and a return to the baseline situation may require more than 3 months of suspension. Galcanezumab was also well‐tolerated with a few AEs, mostly mild. No serious AEs were registered, apart from a transitory ischemic attack, which led to treatment discontinuation (Table 3). The most common AE was constipation, according to the action of galcanezumab, even on the β‐isoform of the CGRP, mainly expressed at the intestinal level (Holzer & Holzer‐Petsche, 2022). Our study has some limitations, such as the lack of a control group and the low sample size. The low number of patients who returned at the control visit after the suspension of galcanezumab did not allow us to make a reliable comparison with the same before suspension. Anyhow, a general worsening was seen. Despite this, the analyzed sample was composed only by severely impaired patients with CM and MOH, followed by a long period of time and this is—to our knowledge—the first study exploring the galcanezumab effectiveness in a sample composed only by CM and MOH sufferers. Our results confirm this drug's effectiveness and safety even in such a severely impaired population.

## AUTHOR CONTRIBUTIONS

Simona Guerzoni and Luca Pani conceived the study. Carlo Baraldi, Flavia Lo Castro, and Maria Michela Cainazzo recruited patients and collected data. Carlo Baraldi made statistical calculations. Simona Guerzoni, Carlo Baraldi, Flavia Lo Castro, Maria Michela Cainazzo, and Luca Pani wrote the manuscript. All authors read and approved the final version of the manuscript.

## CONFLICT OF INTEREST

Simona Guerzoni and Carlo Baraldi received honoraria from Allergan Eli Lilly, Lundbeck, Novartsi and Teva. Flavia Lo Castro received honoraria from Allergan, Lundbeck .and Novartis. Flavia Lo Castro has no conflict of interest to disclose. Luca Pani is the Chief Scientific Officer of EDRA‐LSWR Publishing Company and of Inpeco SA Total Lab Automation Company. In the last year, he has been a scientific consultant to AbbVie, USA; BCG, Switzerland; Boehringer‐Ingelheim, Germany; Compass Pathways, UK; Johnson & Johnson, USA; Takeda, USA; VeraSci, USA; Vifor, Switzerland.

### PEER REVIEW

The peer review history for this article is available at: https://publons.com/publon/10.1002/brb3.2799.

## Data Availability

The dataset analyzed for this article is available from the corresponding author upon reasonable request.
